# Detection of Human Papillomavirus DNA in Patients with Breast Tumor in China

**DOI:** 10.1371/journal.pone.0136050

**Published:** 2015-08-21

**Authors:** Jie Li, Jie Ding, Kan Zhai

**Affiliations:** 1 Department of General Surgery, Beijing Chao-Yang Hospital, Capital Medical University, Beijing, China; 2 Medical Research Center, Beijing Chao-Yang Hospital, Capital Medical University, Beijing, China; Beijing University of Chemical Technology, CHINA

## Abstract

The presence of HPV in breast tissue and the potential causal association between human papillomavirus (HPV) and breast cancer (BC) remains controversial. The aim of the present study was to compare the HPV prevalence in BC tissues, adjacent normal breast tissues and breast benign disease tissues and to investigate the possible association between HPV and breast tumor development in Chinese women. Paraffin-embedded specimens from 187 pairs of BCs including tumor and normal breast tissue adjacent to tumors and 92 breast benign lesions between June 2009 and July 2014 were investigated by nested polymerase chain reaction (PCR) and type-specific PCR, respectively. With strictly quality control, HPV positive infection was detected in three BC tissues. No HPV positive infection was detected in all normal breast tissue adjacent to tumors and benign breast tissues. Through our detailed analysis, rare HPV infection in this study suggests that HPV might not be associated with BC progression.

## Introduction

Breast cancer (BC) is one of the most prevalent malignancies in women, in both the developed and the developing world [1]. It is well recognized as a biological heterogeneous disease in regard to its clinical, histological and molecular profile. Many risk factors have been associated with the pathogenesis of this disease, including family history, hormone levels, cigarette smoking and alcohol consumption. During the past few decades, in addition to known factors, the factor of virus infections has been raised.

Human papillomavirus (HPV) is a small, circular, double-stranded DNA virus that is believed to be an important factor in the pathogenesis of infecting and transforming epithelium to particular benign and malignant lesions in humans. Approximately 120 HPV subtypes have been isolated from humans [2]. High-risk HPV (HR-HPV) encodes a series of proteins, E6 and E7 oncoproteins, that have been associated with cell transformations, which lead to genomic instability that can result in malignancy [3–6]. HR-HPV infection has been thought to be the main cause of human cervical cancers [7], a substantial proportion of other anogenital cancers [8,9], and oropharyngeal cancers [10–12]. Recently, HPV infection is reported to be associated with lung cancers [13]. The first evidence that revealed that HPV might be involved in BC was provided by Di Lonardo et al. in 1992 who demonstrated HPV 16 DNA in 29.4% of paraffin-embedded tissues (PET) of BC by polymerase chain reaction (PCR) using HPV 11, 16 and 18 primers [14]. Many studies have reported HR-HPV infections in BC specimens from diverse populations across the world [15–17]. Koilocyte-like cells were not only observed in HPV positive breast cancer specimens but also observed in some HPV positive normal breast tissue specimens [18]. However, several studies failed to detect HPV in BC tissues [19–27]. Because study designs, involved populations, and HPV detection methods were heterogeneous, the role of HPV in BC remains controversial.

To clarify the relationship between HPV and BC, we performed a case-control study to investigate the presence of HPV in BC tissue, normal specimens adjacent to carcinomas and breast tissue of breast benign disease (BBD) using PCR and analyzed the association between HPV infection and the risk of BC progression in Chinese women.

## Materials and Methods

### Study Subjects

187 sets of BC PET including carcinoma and normal breast tissue adjacent to tumors and 92 BBD were used in this study. Patients were pathologically confirmed without restriction in regard to age and histological type and were consecutively recruited from Beijing Chao-Yang Hospital of Capital Medical University between June 2009 and July 2014. Those who had a history of cancer, metastasized cancer from other organs or neo-adjuvant treatments were excluded. All subjects were genetically unrelated ethnic Han Chinese women. At recruitment, personal data from each participant about demographic information and clinicopathological characteristics were collected. Informed consent was obtained from all participants. The oestrogen receptor (ER), progesterone receptor (PR) and human epidermal growth factor receptor 2 (Her2) statuses of BC patients were also abstracted from the medical records. Written consent had been obtained from every participant. This study was approved by the Institutional Review Board of Beijing Chao-Yang Hospital, Capital Medical University.

### DNA Extraction and Quality Control

Three cuts of 4-μm-thick sections of PET were cut and put into 1.5 ml microtubes. Positive controls (HPV 16 infected SiHa cells and HPV 18 infected HeLa cells embedded in paraffin) and negative controls (blank paraffin block) were also used for quality control. Total DNA from PET was extracted from each subject using the TaKaRa DEXPAT kit (Code No: 9091, TaKaRa, Dalian, China) or Universal Genomic DNA Extraction Kit Ver.3.0 (Code No: 9765, TaKaRa, Dalian, China) according to the instructions of the manufacturer. Amplification of a 268 bp fragment of the β-globin gene was used to assess the quality of DNA in PETs. The primers were GH20 (5′-GAA GAG CCA AGG ACA GGT AC-3′) and PC04 (5′-CAA CTT CAT CCA CGT TCA CC-3′). All samples were positive for β-globin gene, indicating that it was available for the following analysis.

### HPV Detection

Two independent PCRs were applied to detect HPV in tissue. Nested PCR was conducted to amplify fragments in highly conserved regions in the L1 gene. MY09/11 primers were used to amplify 450 bp fragment of L1 region and followed by the secondary PCR using the GP5+/6+ primers to amplify 140 bp inner product [28,29]. Primer sequences were MY09: 5′-CGT CCM ARR GGA WAC TGA TC-3′, MY11: 5′-GCM CAG GGW CAT AAY AAT GG-3′, GP5+: 5′-TTT GTT ACT GTG GTA GAT ACT AC-3′, GP6+: 5′-GAA AAA TAA ACT GTA AAT CAT ATT C-3′. Successfully amplified HPV PCR products were purified and sequenced by an automated sequencer ABI 3730xl (Applied Biosystems, Foster city, CA). The results were be evaluated by NCBI BLAST program. To verify the results independently, we conducted another PCR to amplify the HPV E6/E7 gene of HPV 16 and 18 using type-specific primers (type-specific PCR, TS PCR). The following were primer sequences [30]: HPV16 E6: forward 5'-CTG CAA GCA ACA GTT ACT GCG ACG-3', reverse 5'-CAT ACA TCG ACC GGT CCA CC-3', product of 315 bp; HPV 18 E7: forward 5'-GAG CCG AAC CAC AAC GTC AC-3', reverse 5'-GGA TGC ACA CCA CGG ACA CA-3', product of 152 bp.

### Statistical Analyses

χ^2^ tests were used to examine the deviation of differences in variables. All statistical analyses were performed using Statistical Package for Social Sciences (SPSS) 16.0 for Windows (SPSS Inc., Chicago, IL, USA). A *P* value less than 0.05 was considered significant.

## Results

We examined 279 female breast tumor cases including BC and BBD, aged from 18 to 83 years. Mean age of BC and BBD groups were 57.1 and 36.5 (*P* < 0.001), respectively. The BMI of the BC group (BMI = 24.1) was higher than that of BBD (BMI = 22.3) (*P* < 0.001). The characteristics of the included subjects are summarized in Table 1. Of the total of 279 participants, 148 had invasive ductal carcinoma (IDC), three had ductal carcinoma in situ, five had invasive lobular carcinoma, two had mucinous carcinoma, 29 had unspecified invasive carcinoma, 53 had fibroadenoma, 24 had adenosis, ten had intraductal papilloma, and five had cystic mastopathy. In the BC group, the positive rate of lymph node metastasis, ER, PR and Her2 expression were 38.0%, 72.2%, 69.5% and 86.6%, respectively.

**Table 1:** Clinical and pathological features of paraffin-embedded samples of breast cancer (BC) and breast benign disease (BBD).

Characteristics	BC (n = 187)	BBD (n = 92)	*P*
Age (mean ± SD)	57.1 ± 12.4	36.5 ± 11.8	< 0.001
BMI (mean ± SD)	24.1 ± 4.2	22.3 ± 3.8	< 0.001
Histologic type, n (%)			
Invasive ductal carcinoma	148 (79.1%)		
Invasive carcinoma, unspecified	29 (15.5%)		
Ductal carcinoma in situ	3 (1.6%)		
Invasive lobular carcinoma	5 (2.7%)		
Mucinous carcinoma	2 (1.1%)		
Fibroadenoma		53 (57.6%)	
Adenosis		24 (26.1%)	
Intraductal papilloma		10 (10.9%)	
Cystic mastopathy		5 (5.4%)	
Tumour size			
≤ 2 cm	119		
2-5cm	60		
> 5 cm	8		
Lymph node metastasis(+), n (%)	71 (38.0%)		
Oestrogen receptor (+), n (%)	135 (72.2%)		
Progesterone receptor (+), n (%)	130 (69.5%)		
Her2 status (+), n (%)	162 (86.6%)		

**Fig 1:**
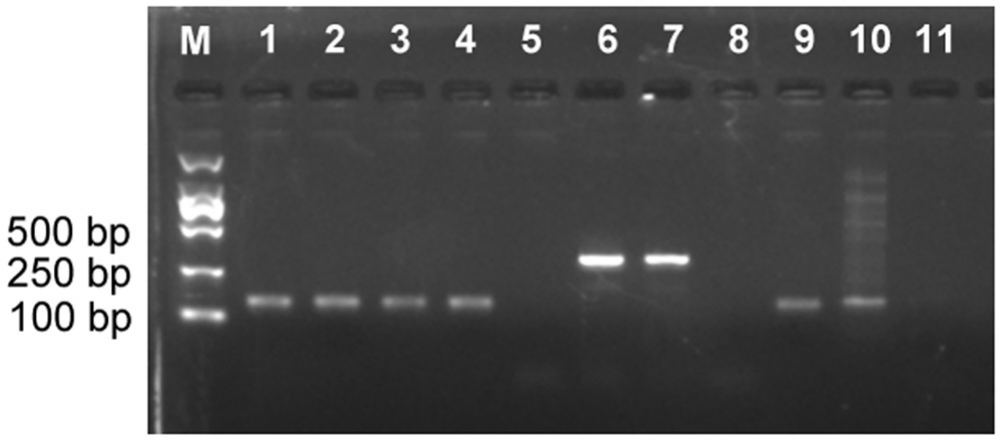
Electrophoresis of representative PCR products of HPV on 2.0% agarose gel. M: Marker (TaKaRa DL2000); Lane1-5: nested PCR products; Lane 6–11: type-specific PCR products; Lane 1, 6 and 9 are positive controls; Lane 5, 8 and 11 show negative controls. Lanes 2–4 show positive bands of 140 bp, Lane 7 and 10 show positive bands of 315 bp and 152 bp, respectively.

Results of HPV-DNA infected status in all specimen were confirmed by PCR using nested PCR and TS PCR, respectively. All samples were positive for β-globin, indicating adequate quality of DNA. The results of HPV positive controls and negative controls revealed that the PCR system worked well, and there was no evidence of contamination. Three out of 187 BC specimen were positive for HPV-DNA. Among them, two IDC were infected with HPV6 and HPV18, respectively; while one unspecified invasive carcinoma was infected with HPV16. However, HPV-DNA was not detected in all normal breast tissue adjacent to tumors and BBD specimen. PCR products of representative samples are shown in Fig 1. The samples were tested twice by different people, and the results were concordant.

## Discussion

Because Band [31] reported that products of HPV 16 and 18 could induce immortalization in human breast epithelial cells and Di Lonardo [14] demonstrated that 29.4% of BC patients had HPV infection, several studies have explored the association of HPV infection and BC risk. If HPV is infected in BC, there should be some differences in HPV infection status in normal tissues adjacent to and in BC in the same patient. The prevalence of HPV existing in BC varies greatly worldwide. Two published meta-analyses show that HPV infection is associated with BC risk [15,16]. Recently, several studies indicated that HPV infection is not associated with BC risk. In this study, to avoid random error of sampling, we detected HPV prevalence in breast tissue from BC and paired normal breast tissue adjacent to tumors, and BBD to investigate the role of HPV infection in breast cancer progression. During HPV infection and integration into the host genome, the L1 gene is frequently lost [32]. Therefore, not only nested PCR of L1 gene but also E6/E7 region TS PCR were performed to detect HPV in the present study. With strict quality control, three HPV-DNA infection was found in 279 participants’ tissues including 187 sets of BC tissue and normal breast tissue adjacent to tumors, and 92 BBD tissues.

Characteristics of the published studies of HPV and BC risk with case-control setting are shown in Table 2 [18,27,33–51]. A literature review of eligible previous studies indicated a high heterogeneity of HPV prevalence in BCs and BBDs across divergent areas that vary from 2.0% to 60.0% and 0.0% to 19.0%, respectively. Another study reported HPV prevalence was highest especially for studies published between 2000 and 2005 (37.28%, 95% CI = 31.67–43.16%) [15]. Most of published studies used PCR based method to detect HPV status in BC tissues, which have a higher rate of false positives. Different positive detection rates and amplification performances were reported in comparing various primer sets [52]. HPV viral load may be very low in breast tissue compared with cervical cancer tissue [53], and sample preparation, DNA isolation and PCR amplification could introduce contamination. All of these factors have been indicated that tissue block section and biopsy preparation may affect HPV detection rates. Because nested PCR is a contamination-prone method, we think that rare HPV infection in our present study is reliable. Three studies that also reported negative HPV infection were conducted in USA, north-west China and Spain. Two of them used TS PCR which is able to test HPV 6, 11, 16 and 18 to detect HPV in PET samples in BC and BBD [33,41], the other used LiPA HPV Genotyping kit [27]. In one study, HPV was detected using ISH in three (10%) BBD samples, but all of the positive specimens became negative after being frozen for 3 months -70°C [41]. In summary, a lack of HPV in both BC and BBD has been reported by three studies using a total of 152 BC and 47 BBD.

**Table 2:** Characteristics of the published studies of human papillomavirus (HPV) and BC risk with case-control setting.

Study	Country	Sample	Detection method	HPV types detectable	HPV types detected	Cases (n = 1833)		Controls (n = 893)	
						HPV (+)	HPV (-)	HPV (+)	HPV (-)
Bratthauer *et al*, 1992 [33]	USA	PET	Type-specific PCR and DB	6, 11, 16, 18	ND	0	28	0	15
Yu *et al*., 1999 [34]	China and Japan	PET	Type-specific PCR and DB	16, 18, 33	33	18	34	1	19
Damin *et al*., 2004 [35]	Brazil	PET	E6 region type-specific PCR and sequencing	16, 18	16, 18	25	76	0	41
Tsai *et al*., 2005 [36]	Taiwan	FF	L1 region MY09/MY11 PCR and SB	Unspecified^a^	Unspecified	8	54	2	42
Choi *et al*., 2007 [51]	Korea	PET	PCR and HPV DNA chip	17 high and 11 low risk types	16, 18, 56, 58, 59, 70	8	115	0	31
de Leon *et al*., 2009 [37]	Mexico	PET	L1 region MY09/MY11 and GP5+/GP6+ or GP5+/GP6+ PCR and sequencing; INNO-LiPA HPV Genotyping kit^b^	Unspecified^c^	16, 18	15	36	0	43
He *et al*., 2009 [38]	China	FF	E6 region type-specific PCR	16	16	24	16	1	19
Heng *et al*., 2009 [18]	Australia	PET	In situ PCR for L1 region of HPV 16 and HPV 18; L1 region MY/GP PCR and sequencing^d^	Unspecified^a^	16, 18	8	20	3	25
Mendizabal-Ruiz *et al*., 2009 [39]	Mexico	PET	E1 region CpI & CpIIG PCR and restriction-pattern typing	6a, 6b, 11, 16, 18, 31, 33, 35, 39	6, 16, 18, 31, 35	3	64	0	40
Mou *et al*., 2011 [40]	China	FF	L1 region MY09/MY11 and GP5+/GP6+ PCR, DB and sequencing	21 types^e^	16, 18	4	58	0	46
Chang *et al*., 2012 [41]	China	FF and PET	E6/E7 region type-specific FQ-PCR; ISH	6, 11, 16, 18	ND	0	48	0	30
Frega *et al*., 2012 [42]	Italy	PET	INNO-LiPA HPV Genotyping kit	28 types	6, 16, 18, 31, 51, 56	9	22	0	12
Glenn *et al*., 2012 [43]	Australia	PET and FF	In situ PCR and E6 region type-specific PCR	NM	18	35	42	11	47
Sigaroodi *et al*., 2012 [44]	Iran	PET	L1 region GP5+/GP6+, CP and FAP primers PCR, respectively, sequencing	Unspecified^a^	6, 11, 15, 16, 18, 23, 124, unknown^f^	15	43	1	40
Liang *et al*., 2013 [45]	China	FF	HC2 High-Risk HPV DNA Test kit^g^	13 high risk types	NM	48	176	6	31
Ali *et al*., 2014 [46]	Iraq	PET	ISH	8 types^h^	16, 18, 31, 33	60	69	3	41
Ahangar-Oskouee *et al*., 2014 [47]	Iran	PET	L1 region MY09/MY11, GP5+/GP6+ PCR and sequencing	Unspecified^a^	6, 11, 16, 35, 52	22	43	0	65
Manzouri *et al*., 2014 [48]	Iran	PET	L1 region GP5+/GP6+ primers PCR and INNO-LiPA HPV Genotyping kit	28 types	16, 18, 31, 33, 35, 45, 11, 43, 44, 55	10	45	7	44
Peng *et al*., 2014 [49]	China	FF	Sequenom MassARRAY^i^	30 types^j^	18	2	98	0	50
Fu *et al*., 2015 [50]	China	PET	E7 region type-specific PCR; ISH	58	58	25^k^/17^l^	144^k^/152^l^	1	82
Vernet-Tomas *et al*., 2015 [27]	Spain	PET	LiPA HPV Genotyping kit^m^	25 types	ND	0	76	0	2
Present study, 2015	China	PET	L1 region MY09/MY11, GP5+/GP6+ PCR and sequencing; E6/E7 region type-specific PCR and sequencing	Unspecified^a^	6, 16, 18	3	184	0	92

Abbreviation: DB, dot blot hybridization; FB, fresh biopsy; FF, fresh frozen; FQ-PCR, fluorescence quantitative PCR; IHC, immunohistochemistry; ISH, in situ hybridization; ND, not detectable; NM, not mentioned; PCR, polymerase chain reaction; PET, paraffin-embedded tissue; SB, Southern blot hybridization.

^a^Detection of PCR product of L1 region could detect a broad spectrum of HPV genotypes.

^b^Commercial assay for HPV genotyping (Innogenetics, Belgium).

^c^Sequencing PCR product of L1 region could detect a broad spectrum of HPV genotypes. INNO-LiPA HPV Genotyping kit could assess 25 HPV types. In this study, nested PCR using MY09/MY11 and GP5+/GP6+ or GP5+/GP6+ alone was performed if DNA quality was good; INNO-LiPA HPV Genotyping kit was used if DNA quality was worse.

^d^In this study, in situ PCR for L1 region, standard PCR for L1 region using MY/GP and sequencing were used to detect HPV types, respectively. To avoid mistakes, we used data that were consistent in these two methods.

^e^DB analysis using the HPV GenoArray Test Kit (Hybribio Ltd, Hong Kong) which could detect 21 types including HPV 6, 11, 42, 43, 44, 16, 18, 31, 33, 35, 39, 45, 51, 52, 53, 56, 58, 59, 66, 68 and CP8304.

^f^A type which could not be genotyped compared to HPV reference sequences.

^g^Commercial assay for HPV genotyping (Digene, Gaithersburg, MD).

^h^This study used two different ISH systems from Zytovision GmbH (detecting HPV 16, 18, 31, 33, 35, 45, 51 and 82) and Maxim Biotech Inc (detecting HPV 16, 18, 31, and 33).

^i^A genotyping platform (Sequenom Inc).

^j^In that study, authors used Sequenom MassARRAY which could detect 21 types including HPV 6, 11, 16, 18, 26, 31, 33, 35, 39, 40, 42, 43, 44, 45, 51, 52, 53, 54, 56, 58, 59, 61, 66, 68, 70, 72, 73, 81, 82 and 89/CP608 to genotype subtypes.

^k^ Results using PCR.

^l^ Results using ISH.

^m^ Commercial assay for HPV genotyping (Laboratory Biomedical Products, Netherlands).

In total, only 7 studies have published HPV status in BC and non-cancer controls with case-control design in female Chinese [34,38,40,41,45,49,50]. In 1992, Yu et al reported 15 of 34 BC infected with HPV 33 and both 2 benign and atypical ductal hyperplasia showed negative HPV infection using TS PCR and dot blot hybridization which could detect HPV 16, 18 and 33 [34]. He et al in 2009 using HPV 16 E6 region TS PCR and Mou et al in 2011 using MY09/MY11 and GP5+/GP6+ primers PCR found that 24 of 50 BC, 1 of 20 normal breast tissue samples and 4 of 62 BC, none of 46 normal breast tissues infected with HPV, respectively [38,40]. Liang et al in 2013 found 48 of 224 and 6 of 37 breast fibroadenomas were HPV infection with hybrid capture 2 assay and no high-risk HPVs prevalence difference were detected between malignant tissues and controls (*P* = 0.468) [45]. Using Sequenom MassARRay platform, Peng et al in 2014 reported 2 of 100 BC and 0 of 50 BBD infected with HPV [49]. Fu et al also compared whether HPV58 present in BC and BBD [50]. However, Chang et al in 2012 reported none of 48 BC and 30 BBD infected with HPV using E6/E7 region TS PCR and ISH [41]. However, only two of these 7 studies using another method to perform the experiment again to verify the results [41,50].

Although HPVs infecting epithelial mucosa or cutaneous surfaces, the possible mechanisms by which HPVs are transmitted to the breast are not clear. HPV virions are known to be released when the envelope of cells desquamate. Therefore, it is assumed that cell surface to surface contact, mainly during sexual activities, play a very important role in HPV transmission [54], as a transmission route of HPV infection in oral carcinogenesis by oral sexual behaviour [55]. Some authors suggested that a haematogenic and/or lymphatic transfer of viruses from one organ to another [56]. Several studies also indicated HPV exist in nipple tissues and breast milk, suggesting that HPV may transfer from nipple to lactiferous ducts [57]. Cells with HPV infection are eliminated by the immune system, and only long-term infections would cause pathogenesis. If HPV plays a role in BC carcinogenesis, HPV would be found in some precancerous lesions or normal tissue. Therefore, it is very important to test HPV status in BC with different stages of progression. Unfortunately, none of the published studies has strictly design to observe HPV infection in BC progression, such as using BC, BC adjacent normal tissue in the same patient and BBD. Although several studies reported higher HPV prevalence in BC compared with BBD, it is too early to conclude that HPV has a causal role in BC.

In summary, the present study using BC, BC adjacent normal tissue in the same patient and BBD examined HPV status in various stage of BC progression, with strictly quality control, we conclude that rare breast tumor infected with HPV and suppose that HPV infection is not associated with BC in Chinese women.
